# Very Elevated IgE, Atopy, and Severe Infection: A Genomics-Based Diagnostic Approach to a Spectrum of Diseases

**DOI:** 10.1155/2021/2767012

**Published:** 2021-09-24

**Authors:** A. Chin, S. Balasubramanyam, C. M. Davis

**Affiliations:** ^1^Baylor College of Medicine, Department of Internal Medicine Houston, Houston, TX, USA; ^2^Baylor College of Medicine, Department of Pediatrics, Section of Immunology, Allergy and Rheumatology, Houston, TX, USA

## Abstract

Elevated IgE has been long recognized as an important clinical marker of atopy but can be seen in a myriad of conditions. The discovery of autosomal dominant STAT3 deficiency marked the first recognition of hyper-IgE syndrome (HIES) and the first primary immunodeficiency linked to elevated IgE. Since then, genomic testing has increased the number of defects with associated mutations causing hyper-IgE syndrome and atopic diseases with *FLG, DOCK8, SPINK5,* and *CARD11,* among others. A spectrum of recurrent infections and atopy are hallmarks of elevated IgE with significant phenotypic overlap between each underlying condition. As treatment is predicated on early diagnosis, genomic testing is becoming a more commonly used diagnostic tool. We present a 6-year-old male patient with markedly elevated IgE and severe atopic dermatitis presenting with staphylococcal bacteremia found to have a heterozygous variant in *FLG* (p.S3247X) and multiple variants of unknown significance in *BCL11B, ZAP70, LYST,* and *PTPRC*. We review the genetic defects underpinning elevated IgE and highlight the spectrum of atopy and immunodeficiency seen in patients with underlying mutations. Although no one mutation is completely causative of the constellation of symptoms in this patient, we suggest the synergism of these variants is an impetus of disease.

## 1. Introduction

IgE, an immunoglobulin produced primarily by B-cells and plasma cells, is an important mediator of allergic disease. Elevated IgE is commonly seen in patients with atopic dermatitis, food allergy, and asthma [1]. Higher, markedly elevated serum IgE above 2000 IU/ml tends to be associated with severe atopy driven by mutations of the epithelial skin barrier or inborn errors of immunity as seen in HIES [1, 2]. Patients with these underlying conditions present with overlapping phenotypes that pose a diagnostic challenge [3]. Early diagnosis and differentiation between these conditions are of priority as management and prognosis can differ. This case highlights clinical and diagnostic considerations when approaching a patient with severe atopic dermatitis and very elevated IgE.

## 2. Case Presentation

A 6-year-old male with eczema since the age of two was hospitalized for an acute eczema flare with low-grade fever and rigors. On arrival, the patient presented with a low-grade fever of 99.4°F with a physical exam notable for diffuse erythroderma with xerosis and open impetiginous lesions throughout the arms, trunk, and face. There were no pustules, vesicles, draining wounds, or extensive denudation. Before this, his eczema had been reasonably controlled with triamcinolone cream and frequent hydration with emollients. There was no history of recurrent viral or fungal infections involving the respiratory or sinus tracts. There was no personal or family history of fractures, skeletal abnormalities, cognitive abnormalities, or vasculitis. Further history elicited no known food allergies in the patient, but an egg allergy in the patient's father.

Initial lab testing showed leukocytosis (14,700 cells/*μ*L) with 27.6% eosinophils and blood cultures positive for methicillin-sensitive *Staphylococcus aureus.* He was treated with intravenous clindamycin and eventually transitioned to oral clindamycin for a fourteen-day course. This initial infection resolved; however, three months later, the patient returned with recurrence of eczematous flares and suspected superinfection. He was treated with a fourteen-day course of oral clindamycin and cephalexin and a five-day course of prednisone. The patient's IgE drawn at this time was 7,460 IU/ml. Food allergen panel was significant for very high specific IgE to peanuts, egg whites, soybean, hazelnut, walnuts, almonds, and cashews. Chest imaging showed no pathology. A hyper-IgE NIH score [3] was 27 (IgE > 2000 = 10 points, eosinophil count >800 = 6 points, mild characteristic facial features = 2 points, severe eczema = 4 points, history of severe nonpulmonary infection = 4 points, and increased nasal width = 1 point).

Despite topical steroids, topical clobetasol, topical tacrolimus, and aggressive skincare, the patient continued to have recurrent eczematous flares associated with cutaneous *Staphylococcus aureus* infection. Prophylactic cephalexin abated some symptoms, and the patient had no additional episodes of bacteremia. Repeat serum IgE level at this time was improved, but still markedly elevated at 3,464 IU/ml. A HIES targeted genetic panel was negative, indicating that the patient had no detectable pathogenic variants associated with *STAT3, DOCK8, TYK2,* and *SPINK5*. Given this negative finding, whole exome sequencing (WES) with chromosomal microarray was performed. Nonsynonymous variants of unknown clinical significance were found in the genes *BCL11B, ZAP70, LYST,* and *PTPRC*, all inherited from the patient's mother, all of which are related to the immune system or immunodeficiency. WES revealed heterozygosity of a pathogenic variant of the filaggrin gene (p.S3247X). The father's WES showed a similar heterozygous mutation in filaggrin despite only reporting alopecia totalis and egg and chicken allergy. Chromosomal microarray analysis showed a copy number gain of chromosome region 15q13.3, which did not contain genes associated with immune function. In addition to prophylactic antibiotics, the patient was started on dupilumab (200 mg every 2 weeks), resulting in fewer eczematous flares with no reported adverse events. At this time, the patient is off of dupilumab, but utilizes ultraviolet therapy every other day, topical steroids, and tacrolimus with nightly wet wraps, and still requires intermittent oral antibiotics for eczema control.

## 3. Discussion

**Figure 1:**
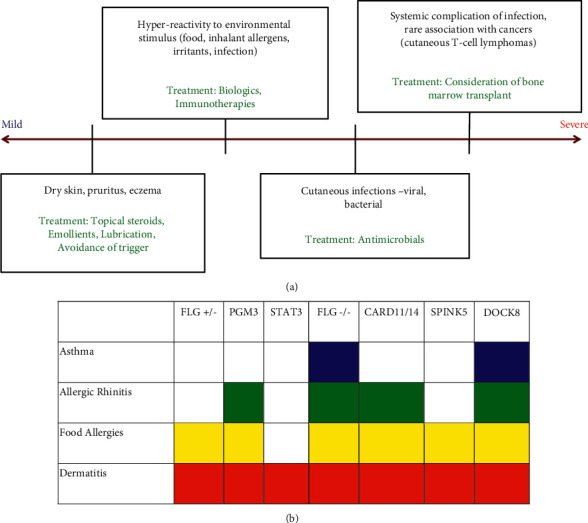
(a) Spectrum of disease in patients with elevated IgE. (b) Genetically defined markedly elevated IgE.

Since its discovery in 1966, IgE has become an important clinical marker of atopy and HIES [1]. Severe atopic dermatitis and HIES can be phenotypically difficult to distinguish clinically as both conditions can present with severe eczematous skin lesions with overlapping infections, markedly elevated IgE (>2000 IU/mL), and eosinophilia [1–4]. The epithelial breakdown that occurs in atopic dermatitis predisposes patients to the atopic march and can become a nidus for infection. This infectious phenotype varies drastically, however, with some patients never experiencing infections and others with persistent skin infections. Mutations involving epithelial barrier proteins, such as filaggrin (*FLG*), *SPINK5, FLG-2, SPRR3,* and *CLDN1*, have become associated with severe atopic dermatitis [5]. Dysregulation of the innate and adaptive immune responses via changes in pattern-recognition receptors, antimicrobial peptides, the Th2 pathway, IL-1, and TSLP have also been linked with atopic dermatitis [5]. HIES, on the other hand, has been generally associated with loss of function in STAT3 protein, resulting in a dysregulated immune response that commonly appears with eczematous skin breakdown and predisposition to sinopulmonary infections [1]. What was once a monogenic syndrome associated with STAT3, HIES has evolved to encapsulate an ever-growing list of gene defects, which are thought to contribute to specific patient clinical features (see Table 1). Currently, the International Union of Immunological Societies has designated pathogenic defects *in DOCK8, SPINK5, PGM3, ZNF341, IL6ST, IL6R, ERBB2IP, TGFBR1, TGFBR2,* and *CARD11/14* genes as causative of HIES [6]. Although each of these genetic deficiencies has unique characteristics, the severity of the disease is based on a spectrum of atopic and infectious features with specific therapeutic implications for the mutations with the poorest outcomes due to severe infection or malignancy (Figure 1(a)). Other primary immunodeficiencies such as Wiskott–Aldrich syndrome (*WAS* gene), IPEX syndrome (*FOXP3* gene), and *TYK2* deficiency also present with eczematous lesions and elevated IgE similar to HIES but are not officially included under the HIES umbrella [1, 4].

### 3.1. Clinical Considerations for Markedly Elevated IgE

Approaching these conditions with understanding the spectrum of atopy may be help parse out key distinguishing features (Figure 1(b)). In STAT3-deficient HIES, IgE is produced in a rather nonspecific manner due to dysregulated immune activation [7]. On the other hand, IgE in atopic dermatitis and DOCK8-deficient patients is more allergen specific. As such, severe FLG-associated atopic dermatitis and DOCK8 deficiency present with more severe environmental and food allergies compared to those with STAT3-associated HIES. In addition, DOCK8 deficiency and FLG null-mutations are associated with early onset asthma [8, 9]. Heterozygous *FLG* deficiency, such as the mutation seen in this patient, is generally associated with milder atopy. The few cases of HIES due to *PGM3, CARD11/14,* and *SPINK5* defects can also present with atopic dermatitis and food allergy [1, 4, 9].

Recurrent skin infections can be seen in almost all conditions associated with elevated IgE; however, the severity, location, and overall burden of infection differ. Patients with atopic dermatitis frequently present with minor superinfections that rarely progress to invasive infection [10]. This patient's staphylococcal bacteremia is consistent with a presentation of STAT3-deficient and ZNF341-deficient HIES, prompting a deeper genetic investigation. This patient had mutations affecting the immune system as described in the next section.

Known genetic defects in HIES, STAT3, and ZNK341 deficiency can be distinguished from other etiologies by the presence of primarily staphylococcal abscesses, boils, and pneumatoceles [3]. DOCK8 deficiency leads to a wide range of severe bacterial and viral infections involving multiple organs [4, 9]. The severity of infections, inflammatory conditions, and occurrence of malignancy in DOCK8-deficient patients results in an increased risk of mortality, as well as significantly decreased quality of life [9]. The recently reported loss-of-function variants in *SPINK5*, *CARD11,* and *CARD14* are not only associated with severe atopic dermatitis but also include sinopulmonary infections [1, 9]. Given these differences, patient management can vary drastically. In STAT3-deficient patients, routine use of prophylactic antibiotics is common, whereas it is generally not recommended in patients with atopic dermatitis [1, 10]. Prophylactic antibacterials and antifungals are also used in DOCK8-deficient patients; however, the severity of infection and degree of immunodeficiency prompt the use of IgG supplementation and hematopoietic stem cell treatment [9].

Clinical scores have been developed to help physicians differentiate HIES from other conditions when genetic testing was not broadly available. The NIH scoring system utilizes 21 clinical and laboratory findings including the presence of increased serum IgE (>10 times normal), eczema, blood eosinophilia, and characteristic facies [11]. In addition, reduced Th17 cell counts can be incorporated for improved sensitivity [11]. Scores ≥40 are considered the cutoff for the diagnosis of HIES, whereas scores below 20 make the diagnosis unlikely. Scores between 20 and 40 result in inconclusive judgments as to the role of the immune system in a patient's presentation, as in this patient. Genetic testing can be helpful in determining how large a role the immune system versus defective barrier function is playing in recurrent infections. Because the sensitivity and specificity of the HIES scores for STAT3-deficient HIES diagnosis have not been fully validated, the NIH scoring system should be used with caution. The presence of food allergies, a feature not included in the NIH-HIES scoring system, is present in up to 85% of DOCK8-associated HIES, but only in 37% of STAT3-deficient HIES [9]. Similarly, unlike STAT3-deficient HIES, PGM3-associated HIES is correlated with increased, not decreased, levels of Th17 [12]. Ultimately, the phenotypes associated with each of these gene defects associated with HIES have a wide range of system involvement that is difficult to capture in a single score (Table 1).

### 3.2. Genetic Considerations for Markedly Elevated IgE

The use of genetic testing is generally recommended in the workup of inborn errors of primary immunodeficiency [2]. Genetic testing of this patient revealed a paternally inherited heterozygous mutation in *FLG* associated with epidermal barrier function and maternally inherited variants of unknown significance (VUS) in *BCL11B, ZAP70, LYST, and PTPRC* associated with immunity. *FLG* defects are classically associated with severe atopic dermatitis; however, the pathogenicity of this patient's heterozygous mutation is less clear [13]. *FLG* mutation carriers tend to have a much milder phenotype [13]. In addition, the type of *FLG* variant may also dictate a level of phenotypic heterogeneity. For example, the p.R501X and p.S761fs genotypes are associated with the most severe disease [14]. This patient's c.9740C > A mutation, otherwise known as the p.S3247X genotype, is a common variant that has a relatively weak association with atopic dermatitis [14].

The discrepancy of this patient's clinical severity with his known *FLG* variant highlights the contributory effect of other genetic mutations associated with elevated IgE or infection [4, 5, 9]. In this case, the patient's father exhibited only mild allergy with no cutaneous symptoms despite having the same heterozygous *FLG* variant as the patient. Studies exploring synergism between multiple polymorphisms are limited, especially when considering the contribution of skin barrier function and immunity. The VUS should be considered when the clinical phenotype of eczema appears out of proportion with known genetic mutations. The *BCL11B* gene has been shown to play a role in epidermal development and is required for the development of *αβ* T-cells and most *γδ* T-cells [15, 16]. Ablation of this protein in mice skin led to enhanced Th2 activation and subsequent development of atopic dermatitis, indicating the role for suppression of Th1 cytokines [16, 17]. Heterozygous mutations in *ZAP70*, a tyrosine kinase expressed on the surface of T-cells, are associated with elevated IgE due to TCR signaling defects and CD8 immunodeficiency due to the termination of thymic development [1, 18]. This patient had mutations in *LYST*, associated with the immunodeficiency Chédiak–Higashi syndrome and *PTPRC*, which encodes CD45 and functions as a signaling gatekeeper in T-cells. All three of the abovementioned genes have been reported to be associated with eczema and immune deficiencies [19]. Given this association with both eczema and impaired T-cell function, there is the potential pathophysiological synergism of all of these variants in this patient, causing severe eczema and recurrent skin infections. This warrants further protein functional testing.

With the advent of next-generation genomic sequencing and molecular diagnostics, multigene panels have emerged as new clinically available tools for physicians. These panels typically include several targeted genes that detect high-yield mutations. For elevated IgE, proprietary panels typically include 3-4 genes that pertain to HIES diagnoses. This can be problematic as many disorders can underlie elevated IgE and may go undetected with these tests. Alternatively, using comprehensive genomic testing with whole exome sequencing (WES) or whole genome sequencing (WGS) offers a less biased approach that may be beneficial in diagnosing patients with specific primary immunodeficiency or patients with an altered immune system predisposing to infection [20]. The diagnostic yield of using these sequencing techniques to uncover subtle or overt immune defects should be further explored in patients with suspected HIES.

## 4. Conclusions

This case highlights the clinical spectrum of atopy and infectious phenotypes among patients presenting with markedly elevated IgE and the potential for synergistic genetic mutations to contribute to severe disease. Ultimately, the use of whole exome sequencing facilitated the diagnosis of *FLG* deficiency and unveiled several additional genetic factors that may contribute to this patient's phenotype through the alteration of his adaptive immunity. With an increasing number of immunologic and atopic diseases associated with elevated serum IgE levels, the definitive diagnosis of these conditions becomes uncertain from a strictly clinical basis. The use of next-generation gene sequencing, choosing targeted gene panels, or whole exome sequencing will need further exploration, but provides an avenue to diagnose patients early in their disease course, offering opportunities for timely intervention.

## Figures and Tables

**Table 1:** Phenotypes of genes involved in patients with markedly elevated IgE levels.

	FLG	PGM3	STAT 3	CARD11/14	SPINK5	DOCK8
Skin	Yes	Yes	Yes	Yes	Yes	Yes
Respiratory	No	Yes	Yes	No	Yes	Yes
Musculoskeletal	No	Yes	Yes	No	No	No
Allergies	Yes	Yes	Yes	Yes	Yes	Yes
Neuro	No	Yes	Yes	No	Yes	Yes
Hem/onc	No	No	Yes	No	Yes	Yes
GI	No	No	Yes	Yes	Yes	Yes

## Data Availability

The patient medical information used to support the findings of this study is included within the article.
